# The occurrence of remitting seronegative symmetrical synovitis with pitting edema (RS3PE) after arthroplasty mimicking a periprosthetic joint infection: A case report and literature review

**DOI:** 10.1097/MD.0000000000040344

**Published:** 2024-11-01

**Authors:** Yoshihiro Araki, Kei Hirose, Maki Hirose, Katsuhiro Hayashi, Satoru Demura

**Affiliations:** a Department of Orthopaedic Surgery, Hirose Hospital, Sabae-City, Fukui, Japan; b Department of Orthopaedic Surgery, Kanazawa University Hospital, Graduate School of Medical Sciences, Kanazawa-City, Ishikawa, Japan; c Department of Surgery, Hirose Hospital, Sabae-City, Fukui, Japan.

**Keywords:** bilateral uni-compartmental knee arthroplasty, periprosthetic joint infection, remitting seronegative symmetrical synovitis with pitting edema

## Abstract

**Rationale:**

As the elderly population grows, the number of joint arthroplasty surgeries is also increasing. Periprosthetic joint infection (PJI) is a postoperative complication that occurs in 1%–2% of the arthroplasties. Once it occurs, PJI is refractory to treatment. Similar symptoms of PJI, including joint synovitis and elevated body temperature, sometimes arise because of crystal arthritis, rheumatoid arthritis, or other inflammatory diseases. Precise diagnosis is essential for determining the optimal treatment strategy.

**Patient concerns:**

An 81-year-old female patient with a history of bilateral knee arthroplasty presented with a high fever of 38 °C and was unable to walk due to swelling and pain in the bilateral lower extremities. Infectious conditions, such as cellulitis or PJI, were suspected. Imaging findings revealed bilateral knee joint synovitis with pitting edema around the lower extremities, and cultures of bilateral joint fluids were negative. No crystals were observed in the joint fluid. Laboratory data revealed highly elevated levels of inflammatory marker; however, antinuclear antibodies, including rheumatoid factor and cyclic citrullinated peptide, were not detected.

**Diagnoses:**

Based on bilateral synovitis with pitting edema in the lower extremities, in addition to negative culture findings and normal antinuclear antibodies, the diagnosis of remitting seronegative symmetrical synovitis with pitting edema (RS3PE) was made.

**Interventions:**

Steroid therapy was performed. The dose was gradually reduced, with the improvement of the symptoms.

**Outcomes:**

The inflammatory reaction promptly decreased and then normalized. With improved inflammation, the symptoms of pitting edema, pain in the bilateral lower extremities, and fluid effusion of the knee joints were reduced. She was able to walk without a cane, and her activities of daily living fully recovered.

**Lessons:**

High fever and synovitis after joint arthroplasty do not necessarily indicate an infectious condition. Clinicians should be familiar with the occurrence of RS3PE, regardless of whether arthroplasty is performed.

## 1. Introduction

Remitting seronegative symmetrical synovitis with pitting edema (RS3PE) is a rare inflammatory disease, and its causes are still unknown.^[1,2]^ Typical symptoms include bilateral pitting edema at the distal extremities (including the upper or lower extremities) and/or fluid collection in the joint due to synovitis, in addition to laboratory findings of highly elevated inflammatory markers and normal antinuclear antibody values. The physical findings of synovitis in RS3PE often resemble those of rheumatoid arthritis, crystal arthritis, or infection such as pyogenic arthritis.^[3–5]^ Thus, the diagnosis of RS3PE must be made prudently after the differentiation of these diseases. However, a previous medical history of bilateral arthroplasties makes it more difficult to obtain a precise diagnosis than in cases with normal extremities because postoperative infection sometimes occurs around the artificial implant.^[6,7]^

Periprosthetic joint infection (PJI) is a complication that occurs in 1%–2% of patients who undergo arthroplasty.^[6–8]^ Although extremely rare, a few case reports have described cases of bilateral PJI.^[9–11]^ Patients with diabetes mellitus, steroid therapy, and those being treated with immunosuppressive drugs such as anticancer agents should regularly monitor the occurrence of infection because of the compromised host. The standard treatment for PJI is additional surgical intervention, including irrigation and debridement, combined with antibiotic administration.^[6,12]^ Thus, early detection of the existence of infection and the subsequent surgical treatment are essential for retaining the primary artificial implant. In contrast, a delayed diagnosis might lead to the removal of the implant, and revision arthroplasty would be required. In addition, long-term antibiotics are necessary for complete recovery from infection.^[6,13–15]^

However, the treatment strategy for RS3PE is completely different from that for PJI. Nonsteroidal anti-inflammatory drugs or steroid administration is standardized for RS3PE therapy. Steroid administration can worsen infection, such as PJI. Thus, a precise diagnosis of RS3PE after differentiating between PJI and other similar diseases is important for optimal treatment. We describe the case of an 81-year-old female patient with bilateral knee joint synovitis with pitting edema around the lower extremities who had undergone bilateral uni-compartmental knee arthroplasty (UKA).

## 2. Case presentation

The patient was an 81-year-old woman with a medical history of bilateral UKA surgeries. She had undergone right-sided UKA 3 years previously, and left-sided UKA 6 months previously. No postoperative complications were observed, and the patient could walk with a T-cane and live a daily life without pain or exercise limitations. The patient had no history of cancer, collagen disease, or diabetes mellitus. She also had no family history of hereditary disease.

She noticed pitting edema and pain in her right lower extremity, with an elevated body temperature of 38 °C. She consulted a treating physician. A laboratory analysis showed moderate elevation of inflammatory markers (C-reactive protein [CRP] of 7.03 mg/dL [reference interval: 0–0.3 mg/dL], white blood cell [WBC] count of 4220/μL [reference interval: 3200–8500/μL], and neutrophil count rate of 62.3% [reference interval: 40%–74%]), and no deep venous thrombosis or slight fluid collection of the left knee joint was observed by ultrasound sonography. Initially, cellulitis was suspected, and oral antibiotics were prescribed. However, the symptoms persisted, and the left-sided lower extremity developed the same condition as the right-sided lower extremity, including pitting edema and pain. She was gradually unable to walk and was admitted 2 weeks after the first notification. Her vitals at admission were stable, with blood pressure of 123/72 mm Hg, heart rate of 80 beats per minute, temperature of 37.3 °C, and oxygen saturation of 96% in room air.

**Figure 1.**
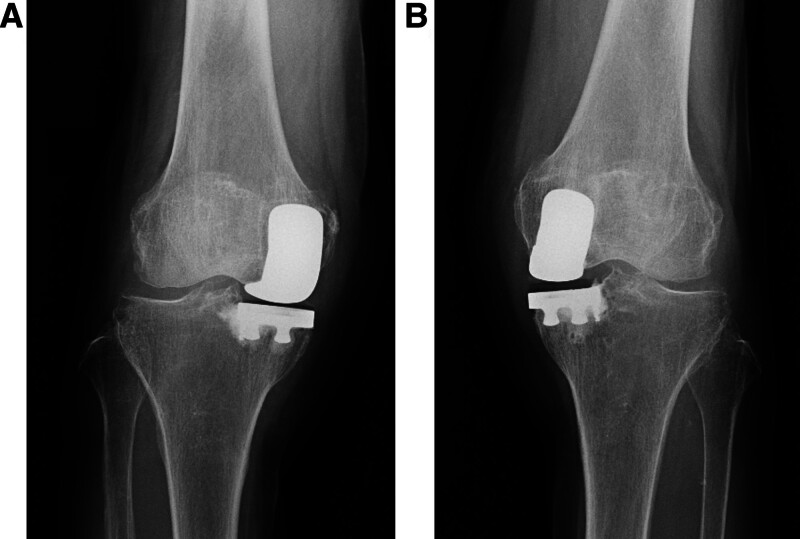
(A) The postoperative X-ray images of the right uni-compartmental knee arthroplasty. (B) The postoperative X-ray images of the left uni-compartmental knee arthroplasty.

**Figure 2.**
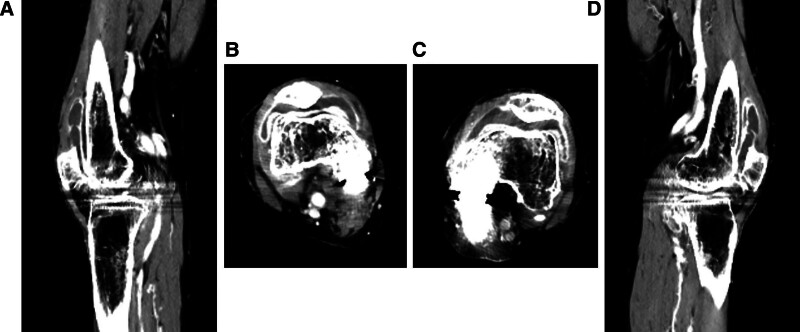
(A) The sagittal images of contrast-enhanced CT for the right knee joint. (B) The axial images of contrast-enhanced CT for the right knee joint. (C) The axial images of contrast-enhanced CT for the left knee joint. (D) The sagittal images of contrast-enhanced CT for the left knee joint.

**Figure 3.**
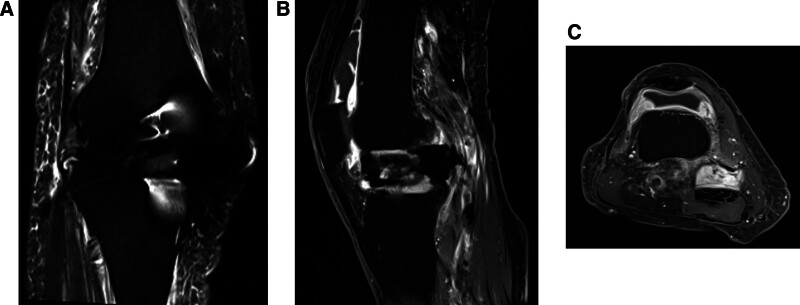
(A) The coronal view of T2-fat suppression MRI for the right knee joint. (B) The sagittal view of T1-weighted enhanced MRI for the right knee joint. (C) The axial view of T1-weighted enhanced MRI for the right knee joint.

No loosening or osteolysis around the implant was observed on radiography (Fig. 1). Bilateral knee joint effusion around the arthroplasty site was observed on contrast-enhanced computed tomography (CT) (Fig. 2A–D). However, the knee joint fluid cultures from both knees were negative, and the blood culture was negative. No crystals, such as calcium pyrophosphate or uric acid, were found in the joint fluid of either knee. Magnetic resonance imaging (MRI) revealed subcutaneous edema and knee joint fluid collection with ring enhancement (Fig. 3A–C). Laboratory data revealed a highly elevated inflammatory markers (CRP of 12.3 mg/dL, WBC count of 4500/μL, neutrophil count rate of 61.3%, and erythrocyte sedimentation rate [ESR] of 117 mm/hour [reference interval: 3–15 mm/hour]), but the serum levels of procalcitonin did not increase, and the autoantibodies, including rheumatoid factor and cyclic citrullinated peptide antibody, were within the normal limits.

**Figure 4.**
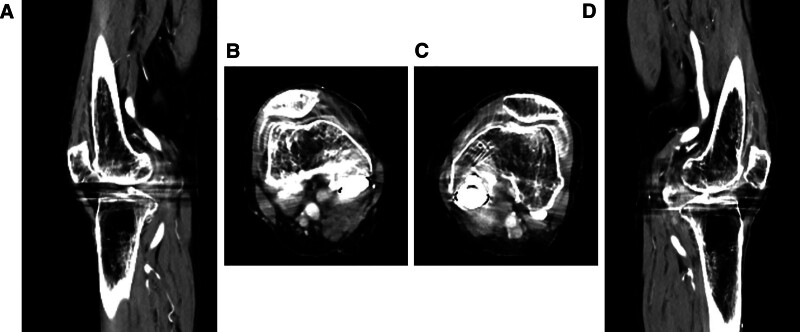
(A) The sagittal images of contrast-enhanced CT for the right knee joint after steroid administration. (B) The axial images of contrast-enhanced CT for the right knee joint after steroid administration. (C) The axial images of contrast-enhanced CT for the left knee joint after steroid administration. (D) The sagittal images of contrast-enhanced CT for the left knee joint after steroid administration.

**Figure 5.**
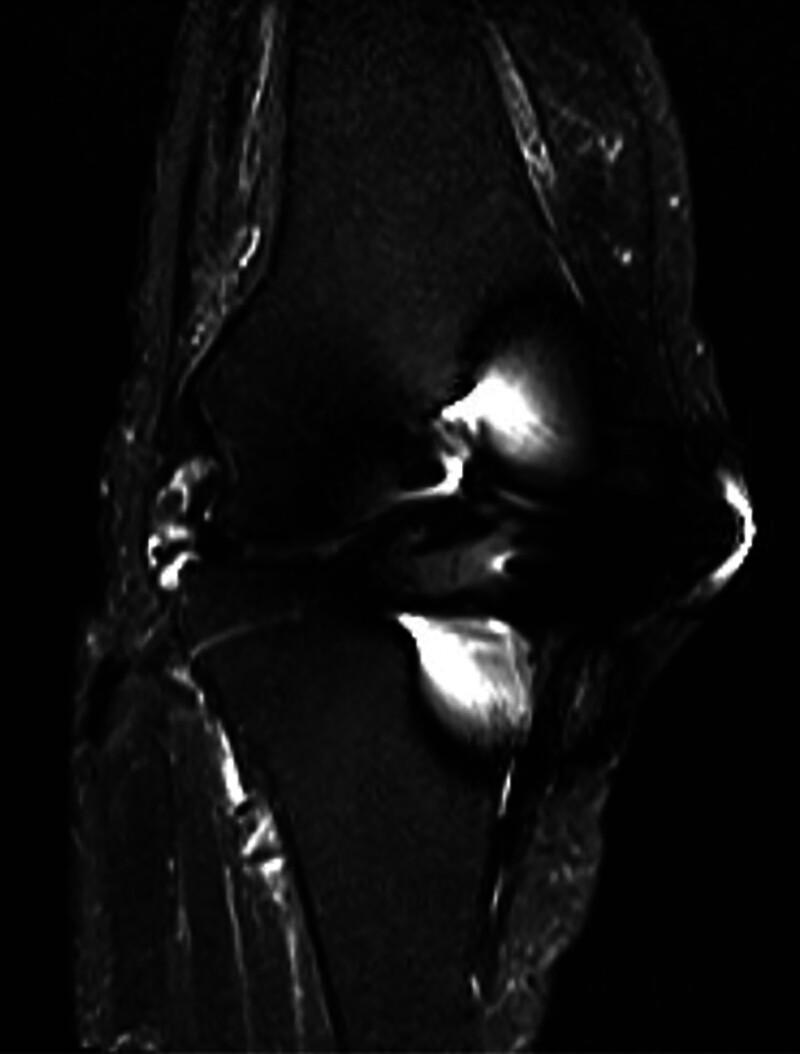
The coronal view of T2-fat suppression MRI for the right knee joint after steroid administration.

After stopping antibiotic treatment, only nonsteroidal anti-inflammatory drugs were administered to relieve pain. The symptoms of pain in the lower extremities gradually decreased; however, the inflammatory reaction persisted. After informing the patient of the risks and benefits of steroid administration and obtaining consent, oral prednisolone was prescribed at a dose of 5 mg/day. The inflammatory reaction immediately decreased, and the symptoms of pitting edema in the lower extremities with joint fluids improved (Figs. 4A–D and 5). At discharge, 2 months after admission, she was able to walk with a T-cane. The administration of prednisolone at a dose of 1 mg/day was continued for approximately 3 months after discharge. No adverse events associated with steroid administration were observed, and the patient’ condition had stayed restored. Thereafter, the patient was free of prednisolone medication.

RS3PE was diagnosed based on physical and imaging findings, and steroid effectiveness. Six months after discharge, synovitis with pitting edema on both sides of the lower extremities was never observed, and laboratory data also revealed no elevation of inflammatory markers (CRP of 0.08 mg/dL, WBC count of 4620/μL, neutrophil count rate of 62.2%, and ESR of 8 mm/hour). At 1 year after discharge, RS3PE showed no signs of recurrence, and the arthroplasty implant survived without any loosening or failure. She was able to fully restore the original life without limitations in activities.

## 3. Discussion

RS3PE is a rare inflammatory disease and treatment with steroids is very effective.^[1,2]^ Thus, an early diagnosis of RS3PE might lead to early improvement of inflammation with relief of symptoms by steroid administration. However, previous surgical intervention for the extremities sometimes makes it difficult to diagnose RS3PE because the symptoms resemble those of postoperative infection, which worsens with steroid therapy.

To the best of our knowledge, there are no previous reports of RS3PE after bilateral knee joint arthroplasty. RS3PE is associated with synovitis of the joints, in addition to pitting edema around the involved joints and extremities. Joint fluids due to synovitis are often observed along with symptoms of swelling, redness, fever, and tenderness. Furthermore, inflammation due to continuous synovitis may cause the activation of osteoclasts, leading to osteolysis, especially in the surrounding bone of the implant for arthroplasty.^[16]^ Aseptic loosening of the implant and failure of arthroplasty may occur owing to the advancement or a long-term duration of osteolysis.^[17]^ Therefore, revision arthroplasty might be required. Thus, inflammation should be promptly decreased according to the causes of joint synovitis, including RS3PE.

Inflammatory synovitis has also been observed in patients with crystals, rheumatoid arthritis, and pyogenic arthritis.^[3–5]^ Thus, differentiation of these diseases is important for the precise diagnosis of RS3PE. Joint fluid culture and examination are indispensable for distinguishing between crystal and pyogenic arthritis.^[4,5]^ Blood tests for autoantibodies are also necessary to distinguish the condition from rheumatoid arthritis.^[3]^ The imaging findings could also help distinguish RS3PE from PJI. Although joint fluid collection with ring enhancement on enhanced CT and MRI is observed in both RS3PE and PJI, the edema of the soft tissues around the joints is predominantly observed in RS3PE, shown as in Figure 3A, and is rarely observed in PJI. However, subcutaneous edema is also observed in the cases of infection (e.g., cellulitis or panniculitis), and this type of edema may rarely occur because of the spread of inflammation from PJI.^[18]^ Thus, the cause of edema must be considered clinically, together with other examination findings, and the diagnosis must not be made based on imaging findings alone.

The exact prevalence of fever in RS3PE is unknown, however a systematic review of 311 cases has estimated the incidence of fever in RS3PE to be 6.3% (21/311).^[2]^ The amplitude of fever was previously reported to range from 37 to 39 °C.^[2,19–21]^ The high fever in the present case made it more difficult to make an appropriate diagnosis because most cases of infectious diseases also present with a high fever. However, fever is also often prominently observed in association with auto-inflammatory disorders.^[22]^ As a result, clinicians should be aware of the fact that RS3PE has the possibility to increase the body temperature due to its association with auto-inflammatory disorders.

PJI is a challenging complication of arthroplasty.^[6]^ A delayed diagnosis and treatment often lead to implant removal and frequently require revision arthroplasty after resolution of infection.^[6–8,12,13]^ Even if an improvement in the inflammatory reaction is obtained and a few negative cultures of joint fluids are confirmed postoperatively, long-term treatment with antibiotics will be also required, and elevated inflammatory marker levels will sometimes persist due to the refractory condition. Thus, most orthopedists may opt to perform urgent surgical salvage interventions when postoperative infection is suspected. If elevated inflammatory marker levels are observed, but bacteria are not detected in the joint fluid, clinicians should carefully consider the patient’s symptoms, refer to imaging findings, and make an appropriate diagnosis (Table 1).

**Table 1:** Summary of clinical characteristics for RS3PR and PJI.

	RS3PE	PJI
Symptoms		
Fever	Rare	+
Joint synovitis	+	+
Swelling	+	+
Redness	±	+
Pain	+	+
Edema of extremity	+	Rare
Bilaterality of symptoms	+	Rare
Laboratory data		
Increased WBC	±	+
Elevated CRP	+	+
Raised ESR	+	+
Increased procalcitonin	‐	±
Joint fluid culture	‐	+
Blood culture	‐	±

CRP = C-reactive protein, ESR = erythrocyte sedimentation rate, PJI = periprosthetic joint infection, RS3PE = remitting seronegative symmetrical synovitis with pitting edema, WBC = white blood cell.

Bilateral PJI is a rare condition that is infrequently reported. It may be observed in the late phases of infection, including sepsis.^[9–11]^ However, the present case did not show septic shock, even though moderately elevated inflammatory marker levels and high-grade fever were observed. PJI could be differentiated based on bilateral symptoms, the fact that the joint fluid cultures were negative for bacteria, and the ineffectiveness of antibiotic therapy.

In the present case, the symptoms of swelling and pitting edema were first noticed in the unilateral extremity, and then spread acutely to the bilateral extremities. Unilateral RS3PE has been also described previously; however, such atypical cases are very rare and were originally inconsistent with the diagnostic criteria for RS3PE, in terms of symmetry.^[23,24]^ This condition may reflect the early phase of the RS3PE. Early diagnosis of RS3PE is important but may be difficult without fulfilling the diagnostic criteria.

Polymyalgia rheumatica (PMR) is an inflammatory disease with symptoms of musculoskeletal pain in elderly people. It is similar to RS3PE and can be effectively treated with steroids.^[25]^ However, musculoskeletal pain in PMR appears mainly at the trunk or proximal extremities, and not at the distal extremities. In addition, joint synovitis or pitting edema is not usually combined. Thus, the physical findings of PMR are definitive to differentiate it from RS3PE.

The recurrence rate of RS3PE is approximately 9%.^[2]^ Recurrence is more frequently observed in patients with malignancies, including cancer, and the disease is likely to relapse after the cessation of steroid therapy. Furthermore, RS3PE has been previously reported to be associated with cancer treatment and cancer itself.^[26–30]^ A questionnaire about previous medical history of cancer can facilitate early diagnosis of RS3PE, and systemic examinations by imaging modalities may lead to the early detection of underlying diseases in cases of refractory RS3PE.

The present study was associated with several limitations. First, nuclear medicine imaging of leukocyte scintigraphy, antigranulocyte scintigraphy, or fluorodeoxyglucose positron emission tomography, which is recently used for the detection of infectious conditions or diseases, was not performed because the imaging inspection is available only in tertiary institutions and must wait a few days or longer for vacant spots.^[31]^ Second, the detection of alpha-defensin in the joint fluid was not conducted because the examination is not covered by medical insurance and is therefore costly, although an acute infection can be detected by neutrophil-releasing antimicrobial peptides.^[32]^ Third, the long-term clinical outcomes were not investigated; thus, further studies with longer follow-up are required.

In conclusion, symmetric physical and imaging findings of synovitis with pitting edema of the distal extremities, laboratory data of highly elevated inflammatory marker levels and normal levels of autoantibodies, negative culture findings, and the absence of crystals in joint fluids are necessary to obtain a precise diagnosis of RS3PE after arthroplasty and facilitate appropriate steroid treatment.

## Acknowledgments

We thank the patient for participating in this case study.

## Author contributions

**Conceptualization:** Yoshihiro Araki.

**Data curation:** Yoshihiro Araki.

**Formal analysis:** Yoshihiro Araki.

**Investigation:** Yoshihiro Araki.

**Methodology:** Yoshihiro Araki.

**Project administration:** Yoshihiro Araki.

**Resources:** Yoshihiro Araki.

**Software:** Yoshihiro Araki.

**Supervision:** Kei Hirose, Maki Hirose, Katsuhiro Hayashi, Satoru Demura.

**Validation:** Yoshihiro Araki, Kei Hirose.

**Visualization:** Yoshihiro Araki.

**Writing – original draft:** Yoshihiro Araki.

**Writing – review & editing:** Yoshihiro Araki, Kei Hirose, Maki Hirose, Katsuhiro Hayashi, Satoru Demura.
